# A retrospective cohort study to evaluate the development of comorbidities, including psychiatric comorbidities, among a pediatric psoriasis population

**DOI:** 10.1111/pde.13772

**Published:** 2019-02-21

**Authors:** Amy S. Paller, Jennifer Schenfeld, Neil A. Accortt, Gregory Kricorian

**Affiliations:** ^1^ Department of Dermatology Northwestern University Feinberg School of Medicine Chicago Illinois; ^2^ DOCS Global Inc. North Wales Pennsylvania; ^3^ Amgen Inc. Thousand Oaks California

**Keywords:** inflammatory disorders, psoriasis, therapy—systemic

## Abstract

**Background/Objective:**

Compared with the adult psoriasis population, knowledge about the incidence of comorbidities in the pediatric psoriasis population is limited. The objective of this study was to assess the prevalence and incidence of comorbidities, including psychiatric comorbidities, in patients with pediatric psoriasis.

**Methods:**

In this claims‐based, retrospective cohort study, patients with pediatric psoriasis were matched 1:3 with a nonpsoriasis cohort based on age, sex, and index date (the earliest of inpatient claims or the latter of two outpatient claims).

**Results:**

Obesity, serious infection, and juvenile idiopathic arthropathy had higher prevalence and incidence rates in the psoriasis cohort than the nonpsoriasis cohort. Psychiatric comorbidities were also more common in the psoriasis cohort than the nonpsoriasis cohort, as were ulcerative colitis and Crohn disease. Stratifying the psoriasis cohort by disease severity—mild and moderate‐to‐severe—found no differences in incidence rates of comorbidities between the two subsets.

**Conclusion:**

The incidence rates of many comorbid conditions were higher for patients with pediatric psoriasis compared with patients without pediatric psoriasis, and similar between patients with moderate‐to‐severe and mild pediatric psoriasis.

## 1 INTRODUCTION

Pediatric psoriasis is a chronic, inflammatory, autoimmune disease characterized by plaques on the skin. Prevalence estimates of pediatric psoriasis (hereafter referred to as “psoriasis”) in the United States range from 0.1% to 1.3%,^1^ with rate increasing linearly with age. While psoriasis has been observed as early as 2.1 months, prevalence rises from 0.1% to 0.5% before puberty to 0.6% to 1.3% after.^1^, ^2^, ^3^

Information about the incidence of comorbidities among pediatric patients with psoriasis in the United States is limited.^4^ In a German study, there was increased risk of hyperlipidemia, obesity, hypertension, diabetes mellitus, and rheumatoid arthritis (RA), and a fourfold increase in the risk of Crohn disease (CD) among children with psoriasis compared with those without.^5^ In U.S. and Danish studies, children with psoriasis were at increased risk for both psychiatric disorders—including depression, anxiety, and bipolar disease—and receiving drugs for psychiatric disorders compared with those without.^6^, ^7^ In this study, we estimated the baseline prevalence, incidence rate (IR), and incidence rate ratios (IRRs) of comorbidities, including psychiatric comorbidities, among pediatric patients with psoriasis and a subset with more severe disease.

## 2 MATERIALS AND METHODS

### 2.1 Study design and patient population

This claims‐based, retrospective cohort study utilized data from the MarketScan^®^ Commercial Claims and Encounters Database between January 1, 2009, and June 30, 2015. The MarketScan Commercial Claims and Encounters Database contains the inpatient, outpatient, and outpatient prescription drug experience of 190 000 000 unique patients between 1996 and 2017 and provides detailed cost, use, and outcomes data for health care services performed in both inpatient and outpatient settings. Two cohorts of patients aged 4 to 17 years were defined—psoriasis and nonpsoriasis. Eligibility for the psoriasis cohort required a confirmed diagnosis of psoriasis, defined as one inpatient or two outpatient claims with an International Classification of Diseases, Ninth Revision, Clinical Modification (ICD‐9‐CM) code of 696.1. Eligibility for the nonpsoriasis cohort required one inpatient or two outpatient medical encounters with no diagnosis of psoriasis or psoriatic arthritis. For both cohorts, the outpatient claims were required to be 30 to 365 days apart; the index date was the earliest of the inpatient claims or the latter of the outpatient claims. The 12‐month follow‐up period began on the index date. A required baseline period was defined as ≥ 12 months of continuous enrollment with pharmacy coverage before the index date. Patients from the nonpsoriasis cohort were matched 3:1 with patients from the psoriasis cohort based on age, sex, and index date (same month and year).

Two subsets of the psoriasis cohort were identified by treatment activity. Moderate‐to‐severe psoriasis was defined as patients without other inflammatory diseases (ie, juvenile idiopathic arthropathy [JIA], psoriatic arthritis [PsA], RA, CD, and ulcerative colitis [UC]) and receiving phototherapy (ultraviolet B [UVB], and psoralen and ultraviolet A [PUVA]) or systemic therapy (conventional synthetic disease‐modifying antirheumatic drugs [csDMARDs; including methotrexate and cyclosporine A], tumor necrosis factor inhibitors [TNFi's], and/or other biologics). Mild psoriasis was defined as patients neither receiving any treatment listed above for psoriasis nor having other inflammatory diseases.

Institutional review board approval and formal consent were not required because patient data were de‐identified by an independent third party before author review, in compliance with Health Insurance Portability and Accountability Act regulations.

### 2.2 Outcome measures

Patients were followed from the index date until the earliest of the following: disenrollment from the database, end of data collection (June 30, 2015), or occurrence of each comorbidity being assessed. Prevalent comorbidities were captured during the baseline period; incident comorbidities were captured during the follow‐up period and excluded prevalent conditions. Comorbidities in this analysis were assessed individually and defined by one inpatient or two outpatient claims; comorbidities were not mutually exclusive, as a prevalent comorbidity did not preclude a patient from being evaluated for another incident comorbidity. A summary outcome—“any comorbidity”—was defined by the number of patients with at least one comorbid condition. Each comorbidity was defined by its specific ICD‐9 code (Supporting Information). In particular, serious infections were defined by a primary diagnosis of the ICD‐9 code “serious infection” for an inpatient hospitalization with at least one overnight stay, as previously described.^8^ Psychiatric comorbidities were defined by one inpatient or outpatient claim accompanied by a pharmacy or medication procedure claim for psychiatric medications (Supporting Information); a summary outcome—“any psychiatric comorbidity”—was defined by the number of patients with at least one psychiatric comorbid condition. Comorbidities were evaluated separately in the moderate‐to‐severe and mild subsets.

### 2.3 Statistical analyses

Descriptive statistics were generated for all baseline data, for example, sample size, percentage, and mean (standard deviation). Prevalence per 1000 patients and IRs per 1000 person‐years with 95% confidence intervals (CIs) were calculated for each comorbidity during the baseline period. IRRs with 95% CIs compared psoriasis and nonpsoriasis cohorts, and moderate‐to‐severe and mild subsets; *P‐*values for IRRs were based on z‐score and calculated using Mid‐P exact test. The mean number of comorbidities and psychiatric comorbidities was estimated among all patients and patients with at least one comorbidity within each cohort.

## 3 RESULTS

### 3.1 Patient disposition

This study included 38 430 patients (N* *=* *7686 psoriasis, N* *=* *30 744 nonpsoriasis). Patients from the two cohorts differed significantly (chi‐squared *P* < 0.0001) in follow‐up time, number of visits to any doctor for any reason during follow‐up, and region (Table 1). Within the psoriasis cohort, 65% of patients were diagnosed by a dermatologist. Within the nonpsoriasis cohort, the most frequent physician specialties were pediatrician, family practice, medical doctor, unknown, and dermatologist. During the baseline period, 4.4% of patients in the psoriasis cohort and 0.2% in the nonpsoriasis cohort had csDMARD exposure; 19.6% in the psoriasis and 7.9% in the nonpsoriasis cohort had oral steroids exposure.

**Table 1:** Patient characteristics during the baseline period

Demographic characteristics	Psoriasis N* *=* *7686	Nonpsoriasis N* *=* *30 744	Chi‐squared *P*‐value
Sex (female), n (%)	4334 (56.4)	17 336 (56.4)	1.0^a^
Age at index date, years, mean (SD)	12.9 (3.2)	12.9 (3.2)	1.0^a^
Follow‐up time, years, mean (SD)	1.9 (1.5)	1.7 (1.3)	< 0.0001
Visits to any doctor for any reason during follow‐up, mean (SD)	20.8 (32.0)	11.3 (18.9)	< 0.0001
Visits to any doctor for psoriasis during follow‐up, mean (SD)	6.1 (15.3)	–	–
Medication use, n (%)			
csDMARDs	340 (4.4)	53 (0.2)	< 0.0001
Oral steroids	4504 (19.6)	2432 (7.9)	< 0.0001
U.S. region, n (%)			
Northeast	1607 (20.9)	5509 (17.9)	< 0.0001
Midwest	1995 (26.0)	7273 (23.7)	
South	2642 (34.4)	11 286 (36.7)	
West	1314 (17.1)	5815 (18.9)	
Unknown	128 (1.7)	861 (2.8)	
Physician specialty,^b^ n (%)			
Dermatologist	5030 (65.4)	n/a	–
Pediatrician	524 (6.8)	n/a	
Medical doctor	419 (5.5)	n/a	
Family practice	421 (5.5)	n/a	
Other	1292 (16.8)	n/a	

csDMARD, conventional synthetic disease‐modifying antirheumatic drug; SD, standard deviation.

a In this case‐control study, patients were matched by gender and age at index; therefore, there is no difference between the patients with pediatric psoriasis and patients without pediatric psoriasis based on these variables.

b Physician specialty refers to the physician who provided the confirmed diagnosis of psoriasis for the inpatient claim or latter of the two outpatient claims. The five most common physician specialties in “Other” included acute care hospital, physician assistant, internal medicine, nurse practitioner, and rheumatologist.

### 3.2 Comorbidities

The prevalence (95% CI) per 1000 patients of any comorbidity was 49.05 (44.22‐54.26) in the psoriasis cohort and 11.94 (10.75‐13.22) in the nonpsoriasis cohort (Table 2). The most prevalent comorbidities in the psoriasis cohort were CD (11.19 [8.95‐13.82]) and PsA (11.19 [8.95‐13.82]) (Table 2), and in the nonpsoriasis cohort were diabetes mellitus (4.46 [3.74‐5.27]) and serious infections (2.99 [2.41‐3.67]).

**Table 2:** Prevalence of any comorbidity, including psychiatric comorbidities

Comorbidity^a^	Psoriasis N* *=* *7686		Nonpsoriasis N* *=* *30 744	
	n	Prevalence^b^ (95% CI)	n	Prevalence^b^ (95% CI)
Any comorbidity^c^	377	49.05 (44.22‐54.26)	367	11.94 (10.75‐13.22)
Inflammatory bowel disease				
Crohn disease	86	11.19 (8.95‐13.82)	19	0.62 (0.37‐0.97)
Ulcerative colitis	23	2.99 (1.90‐4.49)	12	0.39 (0.20‐0.68)
Arthropathy				
Psoriatic arthritis^d^	86	11.19 (8.95‐13.82)	0	0.00 (0.00‐0.00)
Juvenile idiopathic arthropathy^d^	48	6.25 (4.60‐8.28)	18	0.59 (0.35‐0.93)
Ankylosing spondylitis	3	0.39 (0.08‐1.14)	0	0.00 (0.00‐0.00)
Rheumatoid arthritis	3	0.39 (0.08‐1.14)	4	0.13 (0.04‐0.33)
Serious infection	63	8.20 (6.30‐10.49)	92	2.99 (2.41‐3.67)
Obesity	54	7.03 (5.28‐9.17)	66	2.15 (1.66‐2.73)
Diabetes mellitus	44	5.72 (4.16‐7.69)	137	4.46 (3.74‐5.27)
Any cardiovascular				
Hypertension	20	2.60 (1.59‐4.02)	22	0.72 (0.45‐1.08)
Hyperlipidemia	8	1.04 (0.45‐2.05)	15	0.49 (0.27‐0.80)
Congestive heart failure	1	0.13 (0.00‐0.72)	1	0.03 (0.00‐0.18)
Malignancy	10	1.30 (0.62‐2.39)	21	0.68 (0.42‐1.04)
Melanoma	1	0.13 (0.00‐0.72)	1	0.03 (0.00‐0.18)
Nonmelanoma skin cancer	1	0.13 (0.00‐0.72)	0	0.00 (0.00‐0.00)
Lymphoma	2	0.26 (0.03‐0.94)	6	0.20 (0.07‐0.42)
Any psychiatric comorbidity	174	22.64 (19.40‐26.26)	412	13.40 (12.14‐14.76)
Depression	130	16.91 (14.13‐20.08)	313	10.18 (9.08‐11.37)
Bipolar disorder	38	4.94 (3.50‐6.79)	80	2.60 (2.06‐3.24)
Anxiety	35	4.55 (3.17‐6.33)	54	1.76 (1.32‐2.29)
Suicidal ideation	18	2.34 (1.39‐3.70)	50	1.63 (1.21‐2.14)
Substance abuse	6	0.78 (0.29‐1.70)	8	0.26 (0.11‐0.51)

CI, confidence interval.

a Each comorbidity is mutually exclusive.

b Prevalence per 1000 patients.

c Excluding psychiatric comorbidities.

d Psoriatic arthritis and juvenile idiopathic arthropathy were mutually exclusive in this study.

The IR (95% CI) per 1000 person‐years of any comorbidity was 23.64 (21.11‐26.38) in the psoriasis cohort and 12.68 (11.71‐13.70) in the nonpsoriasis (Table 3). The IRs for several comorbidities were significantly higher in the psoriasis cohort than the nonpsoriasis: IRR (95% CI) was 1.86 (1.63‐2.13) for any comorbidity, 1.64 (1.32‐2.05) for obesity, 1.73 (1.38‐2.16) for serious infections, 9.03 (4.94‐16.31) for JIA, and 2.11 (1.31‐3.34) for hyperlipidemia (*P* < 0.05 for all; Table 3). Types of serious infection were not different between cohorts (Table S1).

**Table 3:** Incidence rates and incidence rate ratios of any comorbidity, including psychiatric comorbidities

Comorbidity^a^	Psoriasis N* *=* *7686		Nonpsoriasis N* *=* *30 744		Incidence rate ratio (95% CI) Psoriasis vs nonpsoriasis
	n	Incidence rate per 1000 person‐years (95% CI)	n	Incidence rate per 1000 person‐years (95% CI)	
Any comorbidity^b^	319	23.64 (21.11‐26.38)	633	12.68 (11.71‐13.70)	1.86 (1.63‐2.13)*
Obesity	116	8.05 (6.65‐9.66)	250	4.90 (4.31‐5.54)	1.64 (1.32‐2.05)*
Serious infection	113	7.86 (6.48‐9.45)	232	4.55 (3.99‐5.18)	1.73 (1.38‐2.16)*
Arthropathy^c^	107	7.49 (6.14‐9.05)	17	0.33 (0.19‐0.53)	NA
Psoriatic arthritis^d^	104	7.25 (5.93‐8.79)	0	0.00 (0.00‐0.00)	NA
Juvenile idiopathic arthropathy^d^	38	2.62 (1.85‐3.59)	15	0.29 (0.16‐0.48)	9.03 (4.94‐16.31)*
Rheumatoid arthritis	12	0.82 (0.42‐1.43)	5	0.10 (0.03‐0.23)	8.20 (2.97‐23.96)*
Ankylosing spondylitis	1	0.07 (0.00‐0.38)	2	0.04 (0.00‐0.14)	1.75 (0.16‐19.36)
Cardiovascular	51	3.51 (2.61‐4.61)	136	2.66 (2.23‐3.14)	1.32 (0.96‐1.82)
Hyperlipidemia	28	1.92 (1.27‐2.77)	47	0.91 (0.67‐1.22)	2.11 (1.31‐3.34)*
Hypertension	25	1.71 (1.11‐2.53)	91	1.77 (1.43‐2.18)	0.97 (0.62‐1.50)
Congestive heart failure	2	0.14 (0.02‐0.49)	2	0.04 (0.00‐0.14)	3.50 (0.49‐24.93)
Diabetes mellitus	23	1.58 (1.00‐2.37)	55	1.08 (0.81‐1.40)	1.46 (0.90‐2.39)
Inflammatory bowel disease	17	1.18 (0.69‐1.88)	22	0.43 (0.27‐0.65)	2.75 (1.46‐5.17)*
Crohn disease	14	0.97 (0.53‐1.62)	15	0.29 (0.16‐0.48)	3.34 (1.60‐6.86)*
Ulcerative colitis	9	0.62 (0.28‐1.17)	12	0.23 (0.12‐0.41)	2.70 (1.11‐6.27)*
Malignancy	7	0.48 (0.19‐0.99)	26	0.51 (0.33‐0.74)	0.94 (0.41‐2.18)
Melanoma	0	0.00 (0.00‐0.00)	1	0.02 (0.00‐0.11)	0.00 (0.00‐0.00)
Nonmelanoma skin cancer	1	0.07 (0.00‐0.38)	1	0.02 (0.00‐0.11)	3.50 (0.22‐56.13)
Lymphoma	2	0.14 (0.02‐0.49)	3	0.06 (0.01‐0.17)	2.33 (0.39‐14.01)
Any psychiatric comorbidity	280	19.94 (17.67‐22.42)	851	17.13 (16.00‐18.33)	1.16 (1.02‐1.33)*
Depression	235	16.60 (14.54‐18.86)	707	14.13 (13.10‐15.21)	1.17 (1.01‐1.36)*
Suicidal ideation	75	5.16 (4.06‐6.47)	155	3.03 (2.57‐3.54)	1.70 (1.29‐2.25)*
Anxiety	69	4.74 (3.69‐6.00)	185	3.62 (3.11‐4.18)	1.31 (0.99‐1.73)
Bipolar disorder	41	2.82 (2.02‐3.82)	101	1.98 (1.61‐2.40)	1.42 (0.99‐2.05)
Substance abuse	22	1.50 (0.94‐2.28)	60	1.17 (0.89‐1.50)	1.28 (0.79‐2.10)

CI, confidence interval; NA, not applicable.

a Each comorbidity is mutually exclusive.

b Excluding psychiatric comorbidities.

c Incidence rate ratios could not be calculated for the arthropathy group or psoriatic arthritis because psoriatic arthritis was null in the nonpsoriasis cohort.

d Psoriatic arthritis and juvenile idiopathic arthropathy were mutually exclusive in this study.

* *P* < 0.05.

Because steroid use may increase the risk of serious infections, prevalence and IR of serious infections were determined in patients with baseline steroid exposure. Among those patients, prevalence (21.28 vs 11.10) and IR (10.82 vs 6.03) of serious infections were higher in the psoriasis vs the nonpsoriasis cohort, respectively.

### 3.3 Psychiatric comorbidities

The prevalence of any psychiatric comorbidity was 22.64 (19.40‐26.26) in the psoriasis cohort and 13.40 (12.14‐14.76) in the nonpsoriasis (Table 2). Prevalence of each psychiatric comorbidity is presented in Table 2.

The IR of any psychiatric comorbidity was 19.94 (17.67‐22.42) in the psoriasis cohort and 17.13 (16.00‐18.33) in the nonpsoriasis (Table 3). IRRs for psoriasis vs nonpsoriasis cohorts were 1.16 (1.02‐1.33) for any psychiatric comorbidity, 1.17 (1.01‐1.36) for depression, and 1.70 (1.29‐2.25) for suicidal ideation (*P* < 0.05 for all; Table 3).

### 3.4 Comorbidities by disease severity

In the psoriasis cohort, 1149 (15.0%) patients had moderate‐to‐severe psoriasis. They were treated with phototherapy (46.3%; of whom 96.8% received UVB and 5.6% PUVA), csDMARDs (35.7%), TNFi's (31.0%), and other biologics (4.9%) on or after the index date. They differed from the mild subset in baseline medication exposure (Table S2). IRs for the moderate‐to‐severe subset were highest for serious infections and obesity (Table 4) but were not different from the mild subsets (IRR 0.91 [0.65‐1.27] for any comorbidity, 0.80 [0.45‐1.41] for serious infections, and 1.28 [0.82‐2.01] for obesity; *P* > 0.05 for all). Patients with moderate‐to‐severe psoriasis with steroid exposure had similar serious infection rates to those without steroid exposure (5.42 vs 5.47). IRs were highest for depression, anxiety, and suicidal ideation in both subsets (Table 4). IRs of psychiatric comorbidities were not different between the moderate‐to‐severe and mild subsets, with the exception of anxiety (IRR 1.79 [1.02‐3.13]).

**Table 4:** Incidence rates of any comorbidity, including psychiatric comorbidities, in moderate‐to‐severe and mild pediatric psoriasis

Comorbidity^a^	Moderate‐to‐severe N* *=* *1149		Mild N* *=* *6278		Incidence rate ratio (95% CI) Moderate‐to‐severe vs mild*
	n	Incidence rate per 1000 person‐years (95% CI)	n	Incidence rate per 1000 person‐years (95% CI)	
Any comorbidity*	40	16.14 (11.53‐21.98)	194	17.76 (15.35‐20.45)	0.91 (0.65‐1.27)
Serious infection	14	5.46 (2.98‐9.16)	77	6.82 (5.38‐8.52)	0.80 (0.45‐1.41)
Obesity	24	9.4 (6.02‐13.98)	83	7.33 (5.84‐9.09)	1.28 (0.82‐2.01)
Diabetes mellitus	3	1.15 (0.24‐3.37)	19	1.67 (1.00‐2.60)	0.69 (0.20‐2.33)
Cardiovascular					
Hypertension	5	1.92 (0.62‐4.48)	18	1.58 (0.93‐2.49)	1.22 (0.45‐3.27)
Hyperlipidemia	4	1.54 (0.42‐3.93)	22	1.92 (1.20‐2.91)	0.80 (0.28‐2.31)
Congestive heart failure	1	0.38 (0.01‐2.13)	0	NA	NA
Malignancy	2	0.77 (0.09‐2.77)	3	0.26 (0.05‐0.76)	2.93 (0.49‐17.52)
Melanoma	0	NA	0	NA	NA
Nonmelanoma skin cancer	0	NA	1	0.09 (0.00‐0.49)	NA
Lymphoma	0	NA	2	0.17 (0.02‐0.63)	NA
Arthropathy					
Ankylosing spondylitis	0	NA	0	NA	NA
Any psychiatric comorbidity*	45	18.05 (13.17‐24.15)	216	19.55 (17.03‐22.33)	0.92 (0.67‐1.27)
Depression	37	14.68 (10.34‐20.23)	180	16.18 (13.90‐18.72)	0.91 (0.64‐1.29)
Bipolar disorder	3	1.16 (0.24‐3.38)	35	3.07 (2.14‐4.27)	0.38 (0.12‐1.22)
Anxiety	17	6.57 (3.83‐10.53)	42	3.68 (2.65‐4.97)	1.79 (1.02‐3.13)
Suicidal ideation	11	4.23 (2.11‐7.58)	60	5.27 (4.02‐6.78)	0.80 (0.42‐1.52)
Substance abuse	5	1.92 (0.62‐4.47)	16	1.40 (0.80‐2.27)	1.37 (0.50‐3.74)

CI, confidence interval; NA, not applicable.

a Each comorbidity is mutually exclusive.

Excluding psychiatric comorbidities.

* *P* > 0.05 for all comorbidities evaluated.

### 3.5 Mean number of comorbidities

The mean number of comorbidities (nonpsychiatric and psychiatric) did not differ between patients with and without psoriasis (Table S3).

## 4 DISCUSSION

Although only a limited number of U.S.‐based studies have evaluated comorbidities among patients with pediatric psoriasis, results in this retrospective, claims‐based cohort study found rates of comorbidities, including psychiatric comorbidities, to be consistent with previous studies. Previous studies have reported some association with cardiovascular risk factors, including obesity, hypertension, diabetes, waist circumference, and metabolic syndrome.^4^, ^9^, ^10^, ^11^ Estimates for the prevalence of obesity ranged from 1.8% to 20.2%.^1^, ^12^ We found that obesity and hyperlipidemia were associated with psoriasis based on incidence rates in the psoriasis and nonpsoriasis cohorts; in contrast, we did not find an association between psoriasis and hypertension or diabetes.^5^, ^6^ The number of obese patients in this study is smaller than found in other studies, likely due to the nature of adjudicated claims databases and data collection, which relies on ICD‐9 coding by physicians. In a U.S. study of patients with moderate‐to‐severe psoriasis, 37% were obese; in a German study of patients with mild‐to‐severe psoriasis, 48% were overweight.^4^, ^13^ Obesity may be associated with psoriasis severity, as one study found a greater number of patients with mild psoriasis were overweight and a greater number with severe psoriasis were obese;^4^ we found similar rates of obesity between moderate‐to‐severe and mild patients. Because psoriasis is a risk factor for cardiovascular disease, recent guidelines recommend screening yearly for overweightness, obesity, and hypertension and every 3 years for type 2 diabetes mellitus.^14^

The serious infection rate observed in this study was higher than expected. Incidence rates were similar in moderate‐to‐severe and mild patients but were higher in patients with steroid exposure, which was more prevalent in the psoriasis cohort than nonpsoriasis. In the absence of additional information on the timing between steroid exposure and serious infection, an association between the two is speculative.

The relative risk of incident CD and UC was greater in the psoriasis cohort compared with nonpsoriasis.^15^ Similarly, Augustin et al. found a fourfold increase in CD; however, that study also found a nonsignificant increase in UC in patients with psoriasis.^5^, ^16^, ^17^ Because patients with JIA, PsA, RA, CD, and UC were excluded from the analysis between patients with moderate‐to‐severe and mild psoriasis, we could not assess incidence of these inflammatory conditions in patients by disease severity.

Psychiatric comorbidities in this study were associated with higher IRs in patients with psoriasis than those without, consistent with previous studies. A previous study based on the U.S. MarketScan^®^ database found that depression, anxiety, and bipolar disorder were more common in patients with psoriasis.^6^ In the Danish Civil Registration System, IRs of all psychiatric comorbidities evaluated—alcohol abuse, anxiety, depression, and eating disorders—were higher among patients with psoriasis, although the difference was not significant for anxiety.^7^ Some studies have reported psychiatric disorders to be common in patients with moderate‐to‐severe psoriasis; however, similar results were not found in this analysis.^5^, ^6^, ^18^, ^19^, ^20^ Recent guidelines recommend screening yearly for depression in all patients and for substance abuse in patients over 11 years old.^14^

Findings in this pediatric study are similar to trends for comorbidities in adults with psoriasis. For instance, adult psoriasis is associated with a higher prevalence of diabetes mellitus (1.22, 95% CI 1.11‐1.35) and rheumatologic disease (2.04, 95% CI 1.71‐2.42),^21^ as well as major depression (OR: 2.09, 95% CI 1.41‐3.11),^22^ and an incidence rate per 100 patient‐years of 18.81 for hyperlipidemia, 6.81 for obesity, and 7.36 for depression.^23^

In this study, patients receiving phototherapy or systemic therapy were defined as having moderate‐to‐severe psoriasis. Phototherapy was the most common treatment in this group. Stratifying by disease severity, we found no difference in the prevalence and incidence of all comorbidities assessed. This may be due to the small sample size of the moderate‐to‐severe subset or because patients with moderate‐to‐severe psoriasis, as defined by treatment utilization, were already being treated with systemic treatments and therefore the impact of the disease could not be differentiated from treatment for the disease.

This study has many strengths, including the large population size of the psoriasis cohort. Previous studies of comorbidities in the United States were not based on as large datasets. Each patient with psoriasis was matched to three relatively healthy patients without psoriasis. This study captured a fairly complete list of comorbidities, including psychiatric comorbidities, and included IRs and IRRs of comorbidities among patients with moderate‐to‐severe and mild psoriasis.

Administrative claims analyses involve large cohorts, but lack diagnostic confirmation.^24^, ^25^, ^26^ Pediatric psoriasis may be underdiagnosed by nondermatologists; however, more than 65% of this psoriasis cohort was diagnosed by a dermatologist (Table 1). We utilized a nonpsoriasis control population, whose physician visits may have been related to regular checkups or conditions requiring more frequent visits, allowing the comparison of patients with psoriasis against a general pediatric population. Because this study did not capture data from Medicaid or CHIP programs, the findings are only generalizable to commercially insured populations. Because of the lack of detailed information on disease activity, it was necessary to use treatment as a proxy for disease activity.^6^, ^27^, ^28^ Some pediatric patients may have limited access (or desire) to certain treatments; moreover, physicians may be less likely to initiate therapy with a systemic agent or phototherapy in children than adults; as a result, we may not have captured all patients with moderate‐to‐severe psoriasis. Patients with JIA, PsA, RA, CD, and UC receiving phototherapy or systemic therapy were excluded from both mild and moderate‐to‐severe subsets because we could not distinguish between the conditions for which therapy was prescribed, potentially affecting the results. In addition, IRs of psychiatric comorbidities may have been affected by other confounding factors; however, a broad definition of psychiatric comorbidity was used in this study and IRs are within a similar range as reported previously.^6^ Another limitation of claims‐based data is that we cannot determine why patients received steroids, though likely related to other comorbid conditions such as CD. Further exploration of steroid use in this population is needed. As mentioned above, obesity is likely to have been underreported, considering obesity was captured only by diagnosis codes and not by body mass index levels.^4^, ^6^ Incidence of obesity may also be low because obesity has been found to predate the psoriasis diagnosis in the pediatric population,^29^ resulting in a higher prevalence of obesity in the psoriasis cohort in this study; incident diagnoses of obesity were only captured if developed after psoriasis onset. The reliance of claims‐based data on ICD‐9 coding may also contribute to the underreporting of obesity. Similar to obesity, diagnoses of hypertension, hyperlipidemia, and diabetes mellitus type 2 were based on ICD‐9 codes rather than laboratory values, which were not available in this claims database. Because patients in the psoriasis cohort visited the doctor more often (Table 1), there may be ascertainment bias, as those patients may have been screened more often for comorbidities. Because this study required a 12‐month wash‐out period, if a patient was diagnosed with a comorbidity before that period and again during the follow‐up period, that comorbidity would have been considered incident rather than prevalent; however, this applies to both cohorts. Additionally, comorbidities with a longer latency period (eg, more than 2 years, as for malignancies) may not have been captured during study follow‐up; as both cohorts had similar follow‐up periods, this is unlikely to result in ascertainment bias. Because this study was intended to be descriptive, we were unable to determine whether psoriasis is independently associated with the comorbidities assessed or driven by confounders.

In conclusion, this descriptive study offers more insight, based on a very large sample size, of the association between psoriasis and comorbidities in pediatric patients. The incidence rate ratios of many comorbidities were higher for pediatric patients with psoriasis than those without. Patients with moderate‐to‐severe psoriasis did not appear to have different IRRs compared with those with mild psoriasis. Future queries of databases where disease activity measures are captured would be useful in addressing differences in comorbidities between mild vs moderate‐to‐severe psoriasis, particularly as it relates to obesity and other components of the metabolic syndrome (ie, cardiovascular disease risk factors). Exploration of comorbidities in patients stratified by therapy as well as the dose–response relationship between psoriasis severity and obesity is also needed.

## CONFLICT OF INTEREST

A.S. Paller has been an investigator without personal compensation for AbbVie, Amgen Inc., Celgene, Janssen, Leo, and Novartis; and a consultant with honorarium for Amgen Inc., Eli Lilly, Novartis, and UCB. J. Schenfeld is a contract worker for Amgen Inc. and receives salary from Amgen Inc. through DOCS Global, Inc. N.A. Accortt and G. Kricorian are employees and shareholders of Amgen Inc.

## INSTITUTIONAL REVIEW BOARD APPROVAL

This study did not require approval by an institutional review board because the patient data in this analysis were de‐identified by an independent third party prior to initial review by the authors, in compliance with Health Insurance Portability and Accountability Act regulations. For this type of study, formal consent is not required.
