# Supplementary material for: A retrospective cohort study to evaluate the development of comorbidities, including psychiatric comorbidities, among a pediatric psoriasis population
Source: Pediatr Dermatol. 2019 Feb 21;36(3):290–7. doi: 10.1111/pde.13772 (PMC6593789; doi:10.1111/pde.13772)
Supplement: Supplementary file 1 [file PDE-36-290-s001.docx]

**SUPPLEMENTAL TABLES**

**TABLE S1.** Most Frequent Serious Infections Among Patients in the Psoriasis and Nonpsoriasis Cohorts

**A.** Prevalent Serious Infections

| Serious infection, *n* (%) | Psoriasis  *N* = 63 | Nonpsoriasis  *N* = 98 |
| --- | --- | --- |
| Pneumonia | 10 (10.6) | 12 (9.8) |
| Methicillin-resistant *Staphylococcus aureus* | 5 (5.3) | 4 (3.3) |
| Otitis media | 4 (4.3) | 2 (1.6) |
| Pleural effusion | 4 (4.3) | 2 (1.6) |
| Cellulitis and abscess of foot | 3 (3.2) | 1 (0.8) |
| Urinary tract infection | 1 (1.1) | 7 (5.7) |
| *Clostridium difficile* | 2 (2.1) | 5 (4.1) |
| Viral infection | 1 (1.1) | 5 (4.1) |

*N* = number of patients who had a prevalent serious infection in this study.

**B.** Incident Serious Infections

| Serious infection, *n* (%) | Psoriasis  *N* = 113 | Nonpsoriasis  *N* = 232 |
| --- | --- | --- |
| Urinary tract infection | 13 (8.3) | 17 (5.4) |
| Pneumonia | 12 (7.7) | 26 (8.2) |
| Methicillin-susceptible *Staphylococcus aureus* | 10 (6.4) | 4 (1.3) |
| Cellulitis and abscess of leg | 7 (4.5) | 7 (2.2) |
| Cellulitis and abscess of trunk | 6 (3.8) | 5 (1.6) |
| Septicemia | 4 (2.6) | 15 (4.7) |
| Pyelonephritis | 3 (1.9) | 13 (4.1) |
| Intestinal infection | 0 (0.0) | 12 (3.8) |

*N* = number of patients who had an incident serious infection in this study.

**TABLE S2.** Baseline Medication Exposures in Patients With Moderate-to-severe and Mild Pediatric Psoriasis

| Medication, *n* (%) | Moderate-to-severe  *N* = 1149 | Mild  *N* = 6537 |
| --- | --- | --- |
| csDMARD | 183 (15.9) | 55 (0.9) |
| Oral steroid | 272 (23.7) | 1148 (81.7) |

csDMARD, conventional synthetic disease-modifying antirheumatic drug.

**TABLE S3.** Mean Number of Incident and Grouped Comorbidities

| Description | Patients | Psoriasis | | | Nonpsoriasis | | |
| --- | --- | --- | --- | --- | --- | --- | --- |
|  |  | *n* | Mean (SD) | Median  (min, max) | *n* | Mean (SD) | Median  (min, max) |
| Total number of incident comorbidities* | All patients | 7686 | 0.06 (0.30) | 0 (0, 7) | 30,744 | 0.02 (0.17) | 0 (0, 4) |
|  | Patients with ≥1 comorbidity | 440 | 1.23 (0.58) | 1 (1, 7) | 668 | 1.13 (0.38) | 1 (1, 4) |
| Total number of incident psychiatric comorbidities | All patients | 7686 | 0.06 (0.31) | 0 (0, 5) | 30,744 | 0.04 (0.25) | 0 (0, 5) |
|  | Patients with ≥1 psychiatric comorbidity | 327 | 1.35 (0.69) | 1 (1, 5) | 925 | 1.31 (0.61) | 1 (1, 5) |

*Total number of incident comorbidities excludes psychiatric comorbidities.

SD, standard deviation.
